# Organising for One Health in a developing country

**DOI:** 10.1016/j.onehlt.2023.100611

**Published:** 2023-08-07

**Authors:** Nachiket Mor

**Affiliations:** Banyan Academy of Leadership in Mental Health, India

**Keywords:** One Health, Governance, Public health, Zoonosis

## Abstract

Globally, zoonotic diseases pose an enormous and growing public health challenge, and developing countries like India are at the epicentre of it. Although there is general recognition of this reality, governments around the world have struggled to organise appropriately to respond to it. The widely held view is that organising for One Health requires effective cross-sectoral collaboration, but the prerequisites to enable such collaboration appear almost unattainable. Perhaps an entirely different approach is needed, which is over and above effective collaborations between competing government ministries. The approach would have to recognise that while any organisational response will need to be able to address identified zoonotic diseases and respond effectively to them in times of crises, it would also be required to have the ability to shape the response to *megatrends* such as climate change, deforestation, and the underlying development models of the country. The paper analyses the success and failures associated with the way in which India, Bangladesh, Kenya, and Rwanda have organised for One Health. It also studies the underlying pathways through which zoonotic spillovers take place, and epidemics gather momentum. Based on these critical analyses, the paper concludes that attempts to build single overarching units to address these challenges have only been partially effective. Given the scale and complexity of the challenge, it recommends that, even at the risk of duplication and the very real possibility that unaddressed gaps will remain, an approach, which builds multiple sharply focused units, would have a greater chance of success.

## 1 Introduction

Globally, zoonotic diseases [1] pose an enormous public health challenge. There is also the concern that “the yearly probability of occurrence of extreme epidemics [low-probability-high-intensity events like COVID-19 and the Spanish Flu of 1918 with huge potentials for mortality and significant costs] can increase up to threefold in the coming decades” [2] (square brackets not in the original). In India, each year, zoonoses such as Rabies result in an estimated 20,000 human deaths, while Brucellosis alone causes losses of approximately 30 million man-days with an added economic loss of 240 million (US$ 10.25 million; IMF’s PPP Exchange rate of 23.43/US$, Nov 11, 2022; [3], [4]).

In the past, One Health had been misunderstood as essentially being synonymous with zoonotic diseases transmitted from animals to humans [5], [6]. To develop a deeper understanding of causality and potential solutions, it is now clear that “its viewpoints should move from “proxy for zoonoses”, to include other topics (climate change, nutrition and food safety, policy and planning, welfare and well-being, antimicrobial resistance (AMR), vector-borne diseases, toxicosis and pesticides issues) and thematic fields (social sciences, geography and economics)” [7]. For example, driven by global warming, diseases previously thought to be endemic only in tropical areas, such as Schistosomiasis and Chikungunya, have now been found in Europe [8]. One Health needs to be understood as a broader idea that goes well beyond the narrow and erroneous perspective of zoonosis in which humans are victims in need of protection from animals who are the perpetrators, to one that considers animal, human, and even environmental well-being (beyond health) as being of equal importance. In this conception, direct links between humans and the environment, as in the case of urban areas, would be considered as significant as those between humans and animals.

For example, Hendra virus [9] causes severe disease in humans and horses and has an associated high mortality rate without a known cure [10]. It is traditionally considered a disease in which the Flying Fox, as a healthy carrier of the virus, is a key causal agent [11]. When viewed this way, bats may only be seen as pests. However, a broader perspective suggests that bats, while a proximate vector, with their unique ability among mammals to fly long distances, are an ecologically important species [12]. They have as much of a right to live on earth as humans and are responsible, among other things, for the regeneration of forests [13]. In the normal course, bats, such as the Flying Fox, do not interact much with humans, since they roost in large colonies far from human habitations and feed on nectar in dense forest environments. However, incursions into previously uninhabited forests due to arable farming and the expansion of cities have led to the destruction of these habitats. Rebuilding these habitats and food sources could represent a powerful long-term solution [14] with a positive independent impact on the environment and the bats themselves.

All of this suggests that any effort to organise for One Health at a national or a sub-national level would need to find a way to incorporate these dynamics and complexity while addressing the concerns that arise from human-animal interfaces and the zoonosis resulting from them. There is also concern that even while narrowly addressing zoonoses “the *big politics* of eradicating intermittent disease outbreaks has dominated the approach, with the neglect of a *livelihoods approach* [which is] arguably more pertinent to developing economies or endemic situations” [15] (square brackets not in the original).

This study examines the issue of One Health from multiple perspectives to arrive at an initial framework of how countries can best organise for One Health, specifically attempting to answer the following questions:

1. What should the local and national balance be within such an effort to organise for One Health?
2. Is the effort best centred within the national/sub-national governmental system or outside it?
3. How will the internal sustainability of this effort be ensured while maximising its external impact?

Any organisational solution that emerges needs to improve the country’s ability to address both near-term tactical and long-term strategic issues. Short-term and tactical issues are related to how quickly and well the country is able to respond to challenges, such as large spillovers of diseases from animals to humans. Long-term strategic issues are related to how well the country is able to address phenomena such as climate change and deforestation. Before exploring organizational design solutions, in the following paragraphs, these tactical and strategic perspectives are discussed in some detail so that they may inform subsequent design discussions.

## 2 Preparing for and responding to pandemics in the near-term

There is broad agreement that three critical steps would need to be taken to minimise the likelihood of immediate threats and the risks of larger pathogen spillovers that could overwhelm the human race:

1. an improved ability to “detect and control infectious diseases in farmed animals” [16] and in domestic animals more generally, and, among humans in densely populated urban locations;
2. “better surveillance of pathogen spillover and development of global databases of virus genomics and serology” [17], [18];
3. a substantial reduction in the rate of deforestation and re-afforestation of much of the earth’s surface.

The first two steps require a greater commitment to developing a sound public health response [19], [20] and, with that commitment, can be completed relatively quickly. The third step, despite its critical importance, unfortunately, does not admit of a near-term response but instead requires the longer-term alteration of the country’s core development strategy. This is discussed in the next section on *megatrends*.

In developing a sound public health response, there is a general recognition that there is a need to detect and control infectious diseases both in animals and humans and to develop surveillance to better anticipate the possibilities of pathogen spillovers. However, in responding to outbreaks of zoonotic diseases, the dominant approach has been to focus on human health and emergency response despite the fact that for “some zoonoses, while the human health risks are important, dealing with the animal infection might provide the most effective control route” [21]. For example, in “the 1999 West Nile virus outbreak in New York, veterinarians reported dozens of crows dying some months before the human cases; however, the surveillance network did not clarify who was responsible for investigation of the bird deaths and subsequent communication with public health officials” [21]. In the case of COVID-19, there was concern that domestic pets and animals located in zoos may acquire the infection from humans and, as this study from Australia shows, veterinarians had a particularly key role to play [22]. Reverse transmission from humans to animals is more the exception than the norm, but the case of COVID-19 and its impact on pets shows that it is important to study the dynamics of the disease at the point of origin and not only at its endpoint, in the animal or human patient.

The above discussion suggests that organising for One Health requires effective cross-sectoral collaboration [23], [24], [25], [26]. However, the prerequisites to enable such collaboration appear almost unattainable [6], [27], [28]. Perhaps an entirely different approach is needed, which, while ensuring that important issues are focused on, is less dependent on, for example, the establishment of “mutual trust” between competing government ministries [6]. Therefore, investigating two alternate paths is important:

1. exploring new ways in which collaborations between diverse stakeholders can be facilitated;
2. developing one or more “separating hyperplanes” [29], which allow a clear separation of roles and responsibilities and reduce, if not completely eliminate, the need for coordination. This is for situations in which the costs of coordination exceed the efficiency gains that result from it [30].

## 3 Altering *megatrends* in the longer-term

One Health is complex and enormous issues like climate change, and high rates of deforestation must be incorporated into it. Otherwise, local attempts to implement the One Heath approach will fail because they are likely to be overwhelmed by the sheer power of these *megatrends*. Climate change and rising temperatures accelerate “spread of zoonotic hosts [intermediate hosts of zoonotic pathogens] and vectors”, and “further stimulate the rate of reproduction of both pathogens and vectors” [31] (square brackets not in the original). Deforestation, which is also linked to climate change, results in closer and more frequent contact between humans and wildlife and increases the potential for new viruses to spread throughout the world [32]. Continued deforestation, particularly in the developing world, is a function of a host of other factors, such as low agricultural productivity and population growth.

**Fig. 1:**
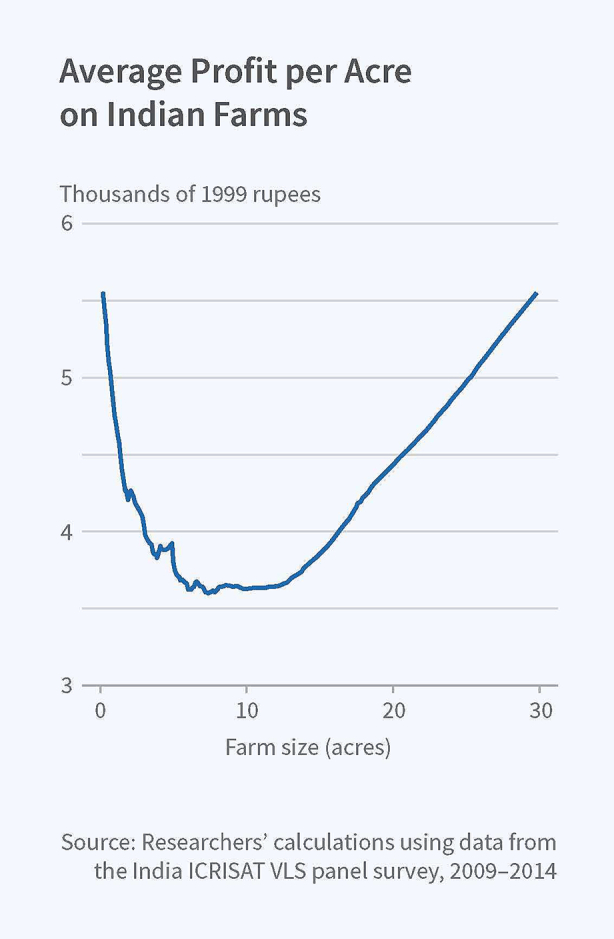
The U-shaped farm productivity curve [33].

On the issue of agricultural productivity, as can be seen from Fig. 1, while there is evidence of a decrease in productivity as farm sizes increase, it is only up to a point after which productivity increases again steeply [33]. There is also strong evidence that, when viewed from the point of labour income (via improvements in labour productivity), the relationship remains linear and positive even with intermediate farm sizes [34]. This suggests that, while there will be a need to address the employment and livelihood needs of the large numbers of people currently working in agriculture, allowing the development of large farms that are highly mechanised could result in a sharp reduction in the quantum of land needed for agriculture without adversely impacting food production or the price of food. It could also allow the introduction of more climate-friendly approaches towards the production of crops such as rice, which, under current production methods, is one of the largest contributors of greenhouse gases [35].

Population growth is driven by increasing fertility trends, which, in turn, is the result of low female literacy rates, high rates of child mortality, and strong “son preference” [36]. An analysis of 2010 data for 131 countries finds that urbanisation makes “an important contribution to fertility reduction and that it influences other factors [in particular education and infant mortality] that encourage fertility decline” [37] (square brackets not in the original). Other studies find similar connections between urbanisation and fertility decline and express the concern that urbanisation is progressing at a much too slow pace in a large developing country such as India, particularly in its poorer northern states [38], [39]. This analysis suggests that, while poorly managed urbanisation produces its own sets of environmental challenges, with the reduction in fertility rates that urban areas engender, the significantly higher population densities they can accommodate, and their higher growth potential, urbanisation can have a strongly beneficial impact on *megatrends* such as deforestation and climate change. The beneficial impact of urbanisation on these *megatrends* could flow through three main channels:

1. with the higher population densities, a reduced need for land for housing;
2. with lowered fertility rates, a reduction in population pressure leading to a decline in the aggregate demand for both housing and food;
3. with higher growth rates, an ability to gainfully employ large numbers of people who may no longer be needed for agriculture.

All of this evidence points to the need for a sustained push toward rapid and high-quality urbanisation [40] and the development of large mechanised farms. The focus needs to be on in situ urbanisation with larger villages turning into cities rather than on large-scale migration to existing cities so that the labour released from agriculture can more easily be absorbed. This could be one of the potential ways forward to stop the continuing deforestation [41], [42] and eventually begin to reverse it.

Whether countries implement the ideas discussed above or develop new ones, approaches to organising for One Health would need to ensure that *megatrends* such as climate change and deforestation receive the attention they require.

## 4 One health country case studies

Several countries have attempted to address the many issues related to One Health by creating suitable organisational structures. Using the examples of India, Bangladesh, Rwanda, and Kenya, the country-level cases of One Health are discussed below. There is much that can be learned from studying their experiences, which can inform future design decisions.

### 4.1 India

**Fig. 2:**
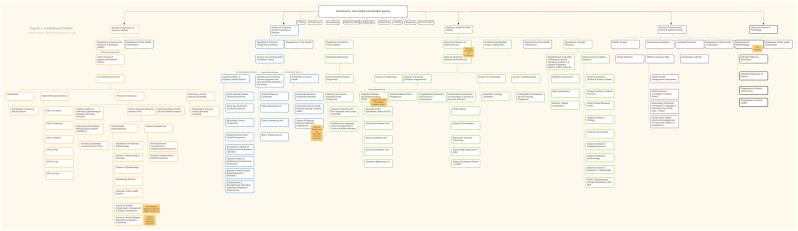
Institutional structures addressing zoonotic diseases in India [5].

**Fig. 3:**
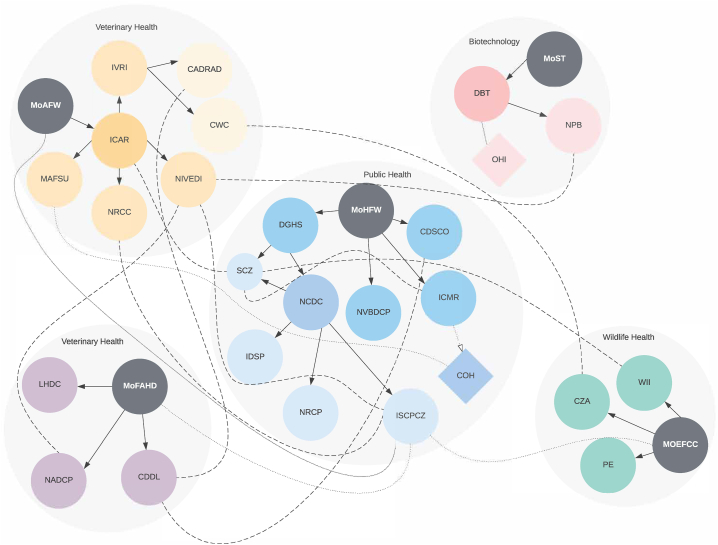
Plot of Institutional Networks Addressing Zoonotic Diseases in India [5].

Paul and colleagues [5] “map existing institutional mechanisms to address zoonotic diseases across the domains of five central ministries [(i) agriculture & farmer welfare; (ii) fisheries, animal husbandry, & dairy; (iii) health & family welfare; (iv) environment, forests, & climate change; (v) science & technology]” (square brackets not in the original) of the Indian government (Fig. 2). They identify “the dissonances and alignment between these ministries and locate existing One Health mechanisms, even if not designated as such” [5]. They find that although the various departments and ministries are “broadly cognisant of One Health principles and that there are a number of inter-ministerial collaborations on zoonoses in India” [5] (as detailed in Fig. 3), they are largely disease-specific and do not involve all relevant sectors in their management. Elimination of human rabies in Goa is a remarkable example of a disease-focused approach that was highly successful [43], which supports the perspective that, while such approaches do not address all aspects of a particular problem, they have the potential to successfully respond to specific threats.

Although Paul and colleagues [5] point to several examples of this lack of intersectoral coordination, one of the most egregious is the complete “absence of the wildlife sector from zoonoses initiatives” despite “well-accepted links between zoonotic diseases in wildlife and humans” [5]. They also expressed concern that even the One Health programs that exist in India suffer from a lack of adequate authority. For example, while the Intersectoral Zoonosis Program “can suggest the involvement of veterinary and wildlife authorities at the state and sub-state levels, it does not have the authority to mandate it” [5] because it is institutionally located within the Ministry of Health & Family Welfare. Paul and colleagues recommend the creation of a “a supra-ministerial One Health mechanism that is not located within or reporting to a single ministry” [5] as a way to address this problem, but do not specify how such a mechanism will exert any authority over the various ministries involved and how funding for it is likely to be found.

In a conversation, Mridula Paul, the lead author of the Paul and colleagues paper [5], shared that while doing fieldwork for her paper, she learned that coordination between various departments improved dramatically at the district level due to the unique role and local authority vested in the District Collector’s office – the District Collector is appointed by the government and is the de facto *CEO* of the district [44]. This observation offers a promising path forward to develop an organisational strategy to respond to pandemics. However, it should be kept in mind that “although the decentralised structure may be appealing in terms of its “power to the people” rhetoric, in the case of infectious disease control, decentralisation has been blamed for a wide variety of inefficiencies within the health and agricultural sectors” [15].

### 4.2 Bangladesh

**Fig. 4:**
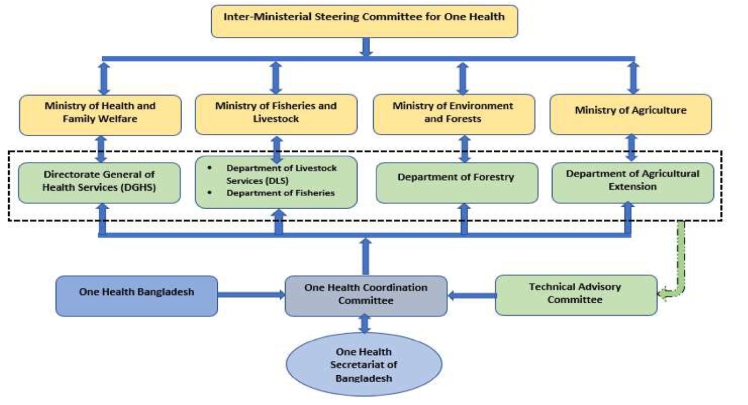
Bangladesh’s One Health governance framework [45].

With a population of over 166 million [46] and a population density of 1,265 people per square kilometre of land [47], Bangladesh is the most densely populated large country (i.e., population > 10 million) in the world. It already has several zoonotic bacterial and viral diseases, including anthrax, leptospirosis, avian influenza, rabies, and Nipah virus [24]. After the outbreak of the avian influenza virus in 2007–2008 in both poultry (H5N1) and humans (high pathogenic avian influenza —- HPAI), Bangladesh established One Health Bangladesh as a “national-level professional organisation” [24] with the “Institute of Epidemiology and Disease Control and Research (IEDCR) with the Directorate General of Health Services in the Ministry of Health and Family Welfare, and the Epidemiology Unit in the Department of Livestock Services within the Ministry of Livestock and Fisheries” as its founding members [48]. In 2012, the ministries of Health and Family Welfare, Fisheries and Livestock, and Environment and Forest, “supported the development of the “Strategic Framework for the One Health Approach”, a guideline for implementing the One Health approach in Bangladesh” [24]. This led to, in 2016, the creation of an Inter-Ministerial Steering Committee for One Health (IMSCOH) and the establishment of a One Health Secretariat at IEDCR with seconded officers from the ministries of health, fisheries, environment, and agriculture (Fig. 4). The One Health Secretariat has been very effective in organising consultations between relevant stakeholders and training programs, and in coordinating outbreak investigations, but has faced challenges in addressing the different objectives and lines of authority of its staff members, as they belong to different ministries [45].

### 4.3 Rwanda

Given its unique history, geography, and economic situation, Rwandans have long been aware that addressing the multiple problems they face depends on “interdependent systems, shared responsibility, involvement of the community, and collaboration across government agencies, content specialists and policies” [49]. Driven by this experience, the Rwandan government has, therefore, “framed policies and priorities to drive toward an integrated, holistic-system approach” [49] even for One Health. With this in mind, in 2015, the Rwandan government approved a One Health Strategic Plan intended to meet the following broad goals (drawn from [49]:

• Promote integrated disease surveillance, prevention, and response (animals, humans, and agriculture);
• Improve education and communication among animal, human, and environmental professionals;
• Expose and integrate students engaged in professional education at the university level to concepts related to One Health;
• Promote interprofessional collaboration around innovation, research, and discovery;
• Develop educational tools for preuniversity education that introduces concepts of One Health;
• Develop policy focused on upstream drivers of disease emergence, including land use, water access, and deforestation;
• Address issues related to land use planning, reducing contact between humans, domestic, and wildlife with minimal changes to critical habitat; and
• Address nutritional access by developing safer practices related to bush meat and animal consumption.

**Fig. 5:**
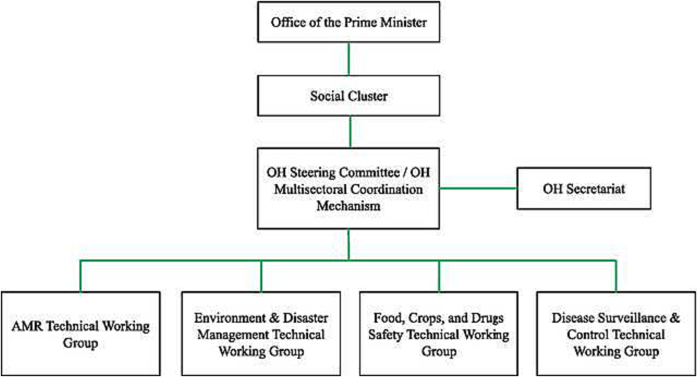
Rwanda’s One Health governance framework [50].

Fig. 5 describes the key components of the One Health governance framework in Rwanda. It is overseen directly by the prime minister’s office (PMO) with a “social cluster”/“One Health Multisectoral Coordination Mechanism (OH-MCM)” [50], comprising the ministers of health, agriculture and animal resources, emergency management, environment, and education, approving the policies and action plans, and reviewing progress periodically. A small One Health Secretariat (one program manager, one administrator, three One Health professionals) assists the OH-MCM while specialised technical working groups “provide expertise on different OH issues including antimicrobial resistance (AMR), zoonotic diseases and OH workforce development, and assist the OH-MCM by managing the implementation of thematic and technical activities of the OH Strategic Plan” [50].

Rwanda has built a network of well-equipped laboratories to assist in its surveillance activities, which are coordinated by the National Reference Laboratory (NRL) for human health and by the Central Laboratory of the Rwanda Agriculture Board and the Rubirizi National Veterinary Laboratory for animal health. And, while its animal health surveillance “is not electronic and is based on annual disease detection studies of zoonoses” [50], it has, among others, an electronic Infectious Disease Surveillance Response (IDSR) system that is deployed in both “public and private health facilities, to support early detection, reporting, tracing, and response to infectious diseases” [50]. In addition, Rwanda has sought to leverage “its decentralized network of community health workers, community-based animal-health workers, healthcare facilities, park rangers, border agents, farmers, and domestic-animal owners as sentinels for monitoring potential zoonotic disease outbreaks” [51]. Furthermore, in pursuit of its goals of introducing these concepts early, “OH has been integrated into curricula at the University of Rwanda’s undergraduate courses and in the medical and Master’s in Global Health Delivery program at the University of Global Health Equity” [50].

However, although “Rwanda has advanced OH through policies, strategies, and structures that promote collaborations across sectors and disciplines” [50], as in the case of other developing countries, it faces severe financial and human resource constraints. Furthermore, in the absence of accountability mechanisms, the ministries continue to work in silos, as do the human and animal health laboratories. And, while the positioning of the OH-MCM within the PMO does signal its importance, since it has no formal position within the government structure, unsurprisingly, it has had a limited impact even within its constituent ministries [50].

### 4.4 Kenya

Kenya created a Zoonotic Disease Unit (ZDU) in 2012, “to establish and maintain active collaboration at the animal, human, and ecosystem interfaces towards better prevention and control of zoonotic diseases” [52]. It was established with a medical epidemiologist deployed by the Ministry of Health (MOH) and a veterinary epidemiologist deployed by the Ministry of Agriculture, Livestock, and Fisheries (MALF). Both epidemiologists deployed to the ZDU remained part of their respective ministries, and the ZDU served as the secretariat of a multisector zoonosis technical working group that provided guidance and leadership to the government on the prevention and control of zoonoses. In 2013, the ZDU began the process of establishing county and sub-county One Health systems by appointing and training One Health persons from each of these levels. In addition, an environmental ecologist was added to the permanent staff of the ZDU [52].

It also developed a 5-year plan for the implementation of OH in Kenya with three objectives (drawn from Mbabu and colleagues [52]).

1. Establish coordination structures and partnerships that promote OH in the country. While the human-animal health link is evident in the organisational structure of ZDU, participation of other areas, including the environmental sector (entomology, microbiology, meteorology, geology, ecology), is important in understanding the factors associated with endemic and emerging disease threats. Through the ZDU, the links between national and subnational human and animal health activities will be enhanced. Furthermore, the curriculum of medical, veterinary, and public health institutions will be revised to include OH approaches. The ZDU will create OH structures at the county and sub-county levels, involving the identification and training of the OH officer of animal or human health within each of Kenya’s 47 counties.
2. Strengthen the surveillance, detection, prevention, and control of zoonoses in humans and animals. Kenya plans to strengthen the systematic surveillance of zoonotic diseases in animals and humans to understand the burden of disease and identify hot spots within the country. Subsequently, the country will develop or adopt prevention and control guidelines for each disease, including supporting the testing and licensing of approved and commercially available animal and human vaccines for the prevention of zoonotic diseases.
3. To stimulate and conduct research and training at the human-animal-ecosystem interfaces. In addition to identifying and promoting priority research on zoonoses, the ZDU provides field training and mentorship to veterinary, medical, and public health trainees using existing surveillance and training platforms. Special studies will be carried out to understand the socioeconomic impact of zoonotic diseases on individual households and the country. During zoonotic epidemics, the ZDU will conduct special studies to determine transmission mechanisms, including subtypes of cross-species pathogens.

The ZDU has enjoyed considerable success despite its relatively lean staffing and is now fully integrated as a functioning unit by both ministries (MOH and MALF). In terms of its work, in addition to the five-year plan mentioned above, the ZDU developed a list of “zoonotic diseases of importance in Kenya” [53]. The prioritization of these diseases was then “carried out through a facilitated consultative process involving 36 experts in zoonoses from the public health (n  = 19), animal health (n  = 15) and wildlife health (n  = 2), during a three-day workshop in September 2015” [53]. Through this process, an orderly set of prioritisation criteria (Table 1) was developed and, using those criteria, a list of priority zoonotic diseases (Table 2) were created. The ZDU was also able to create a risk map and a contingency plan for the rift valley fever disease, and a strategic plan for the elimination of rabies in the country [52].

**Table 1:** Criteria for prioritisation (adapted from Mbabu and colleagues [52]).

#	Description
1.	Emerging or re-emerging disease
2.	Epidemic potential
3.	Severity of disease in humans
4.	Public health emergency of international concern (PHEIC)
5.	Ease of animal-to-human transmission
6.	Ease of human-to-human transmission
7.	Socio-economic implication
8.	Potential for use in bioterrorism
9.	Inadequate knowledge of the disease in the country
10.	Difficulty in management of disease in animals and/or humans
11.	Lack of diagnostic and intervention capacities
12.	Possibility of rapid health gains following public health activities

**Table 2:** Priority zoonotic diseases for Kenya (adapted from Mbabu and colleagues 52]).

Disease Category	Criteria for Prioritization
Viral hemorrhagic fevers:	1 to 10
Crimean-Congo hemorrhagic fever, Dengue, Rift Valley fever	
Yellow fever, Ebola, Marburg	
Avian and other pandemic influenza	1, 2, 3, 4, 5, 6, 7, 9, 10, 11
Brucellosis	5, 7, 8, 10, 12
Leishmaniasis	3, 10, 11
Leptospirosis	2, 9, 10, 11, 12
Anthrax	2, 4, 8
Rabies	3, 12
West Nile	1, 2, 9, 11
Bovine tuberculosis	3, 9, 10, 11, 12
Plague	2, 4, 8, 9
Tularemia	8, 9, 11
Protozoan infection:	9, 10, 11
Cryptosporidiosis, Toxoplasmosis	
Salmonellosis	2, 3, 6, 12
Helminthiasis:	7, 9, 10, 11, 12
Trichinosis, Cysticercosis, Echinococcosis (Hydatidosis)	
Sarcopsis (Mange), Diphyllobothrium	
Fungal infection:	9, 10, 11, 12
Dermatophylosis, Histoplasmosis, Cryptococcosis	
Aspergillosis	
Schistosomiasis	7, 12
Trypanosomiasis	3, 7, 10, 12

If the only interest is in the transmission of diseases from animal hosts and reservoirs to humans, the ZDU provides a good model “for cooperation between human and animal health sectors at a national level” [52], but the approach will need to be considerably broadened to include, for example, environmental risks and move beyond the national level to the sub-national level [54], [55].

## 5 Separating hyperplanes

From the above discussions, it is clear that while there are interesting models to consider for narrow and well-defined responses to One Health crises, a stable response to address the long-term issues related to One Health has proved elusive [57], [58]. “The argument for inter-ministerial platforms to coordinate policy and action for zoonoses control is well founded. However, while One Health is theoretically and, arguably, economically attractive, significant political will and state capacity are required to overcome existing institutional [particularly those relating to inter-ministerial coordination] and financial barriers to its implementation; particularly in developing countries where numerous health and development priorities compete for attention and programmatic funding” [59] (square brackets not in the original). In the context of climate change, “cap and trade” [60] has provided a partial solution, which is beginning to exert pressure on consumers and greenhouse gas producers to begin to reduce them, using differential prices as the tool or “separating hyperplane” [29], making it unnecessary to develop complex coordination mechanisms once the cap-and-trade regime has been implemented [61]. In this section, we explore whether a similar reduction in complexity is possible in the One Health context.

In their work Loh and colleagues [56], find that “the major transmission pathways for zoonoses differ widely according to the specific underlying drivers of EID [emerging infectious disease] events (e.g., land-use change, agricultural intensification). These results can be used to develop better targeting of surveillance and more effective control of newly emerged zoonoses in regions under different underlying pressures that drive disease emergence” (square brackets not in the original). Given the salience of some of these drivers, is it possible to develop strategies that reduce the dimensionality of the problem? Instead of seeking to develop one overarching operating unit for One Health within, for example, the health ministry, would establishing discrete units within multiple ministries which have a narrower focus on disease drivers associated with the primary tasks of that ministry reduce the need for coordination?

Table 3 gives the primary transmission pathway associated with each pathogen. Table 4 gives the most important transmission pathways associated with each EID event. Table 5 maps Table 3, Table 4 onto each other and attempts to identify the pathogen that would be the most of concern for each EID event. From Table 5, a potential approach to organising for One Health that emerges is to build a unit specialising in that particular pathogen within the ministry that is most responsible for that EID event. The predominant pathogen associated with each EID event identified in Table 5 would suggest that, for example, it would be useful to build a One Health unit focused on Rickettsial & Protozoal diseases within the Ministry of Housing and Urban Affairs, Bacterial diseases within the Agriculture Ministry, and Viral diseases within the Ministry of Travel & Tourism. This taxonomy is merely by way of an example of how one might build multiple sharply focused units which align with the core mission of the concerned ministry. This taxonomy is derived from an understanding of the principal transmission pathways associated with specific classes of pathogens using the work of Loh and colleagues [56]. There could potentially be other ways of providing focus and minimising overlap, which a more detailed discussion could arrive at.

**Table 3:** Transmission Pathways by Pathogen (adapted from Loh and colleagues [56]).

**Table 4:** Transmission Pathways by Disease Driver (adapted from Loh and colleagues [56]).

**Table 5:** Disease Drivers & Pathogens (combining Tables 3 & 4).

## 6 Discussion

In working toward the desired national organisation structure for One Health, there are several lessons, set out in Table 6, that can be gathered from the experiences of India, Bangladesh, Rwanda, and Kenya and the “Transmission Pathway” Framework (TPF) drawn from [56]. Three potential ideas related to the organisation of One Health emerge from the lessons outlined in Table 6.

**Table 6:** Lessons for the Organisation of One Health.

Source	Less Promising Ideas	More Promising Ideas
India	Locate the entire One Health programme within a single ministry such as the National Ministry of Health.	Take advantage of historical governance structures such as that of the District Collector in India to ensure ground-level intersectoral collaboration during a crisis bearing in mind that even with this District Collector system, there is a need for coordination at the national level, especially when there are crises spreading across multiple districts that would need to be factored in.
Bangladesh	Locate the entire One Health programme within a single ministry such as the national Ministry of Health.	Set up a lean independent technical unit with personnel drawn from a few ministries who remain on the staff of their respective ministries; build a professional cadre of One Health professionals and professional bodies to steward and promote the growth of these professional capacities.
Rwanda	Locate units responsible for operational coordination outside line ministries even if they report to the Prime Minister or the Chief Minister.	Invest early in building a network of well-equipped laboratories to assist in surveillance activities for both human and animal health; leverage a wide range of front-line workers going beyond community health workers and healthcare facility-based personnel to include community-based animal-health workers, park rangers, border agents, farmers, and domestic animal owners as sentinels for monitoring potential zoonotic disease outbreaks; integrate One Health into university curricula at the undergraduate level as well as in graduate-level medical and public health courses.
Kenya	Expect a lean technical unit to address broader and longer-term concerns related to One Health.	Set up a lean independent technical unit with personnel drawn from a few ministries who remain on the staff of their respective ministries; ensure that the budget of the technical unit features clearly within the budgets of each of the ministries; establish clear prioritisation criteria for identified diseases. In this context, Tanzania’s One Health Coordination Desk (OHCD), set up in the PMO, is also a good example of effective coordination and implementation of One Health policies [62].
Transmission Pathway Framework (TPF)	Expect the TPF framework to be comprehensive.	Establish autonomous units within each ministry to focus on the principal pathogens and transmission pathways that come predominantly within its purview.

The first one relates to establishing one or more national-level extra-ministerial units which operate with an independent budget and authority and are tasked with the responsibility of framing a broader One Health direction and agenda for the whole country. These units also have the responsibility of coordinating with similar efforts around the world. More concretely, in the Indian context, a possible idea would be to create a One Health Science Unit within the office of the Principal Scientific Advisor to the Prime Minister that focuses on ensuring that the country has the scientific, laboratory, and surveillance capacity to identify emerging pathogens. This unit could also coordinate with the Education and Health Ministries to ensure that One Health is prominently featured in both undergraduate and graduate curricula. Additionally, a One Health Strategy Unit could be created within NITI Aayog with the responsibility of ensuring that the overall longer-term development strategy of the country is consistent with minimising One Health risk so that the *megatrends* mentioned earlier can be addressed effectively.

The second relates to using the TPF to identify the most appropriate line ministries and establishing a One Health unit within each one with a clearly and narrowly defined mandate that captures most of the One Health risk drivers that come under the purview of that ministry. In the Indian context, such units could potentially be established within the Ministry of Agriculture to address bacterial pathogens; the Ministry of Housing and Urban Affairs to address rickettsial and protozoal pathogens; and the Ministry of Travel & Tourism for viral pathogens. Such an approach would require ensuring that there is adequate technical capacity within each unit. Absent this capacity, the unit could fail to discharge its responsibilities and potentially miss key signals related to zoonotic spillovers. In the Indian context, this risk is low because most ministries and their counterparts at the state level already have substantial healthcare-related capacities – urban municipalities, for example, already manage large health systems.

As discussed earlier, the risk of a distributed approach is that there could be important coordination failures at critical junctures. To mitigate this risk and to maintain an overall view of the evolving scenario in the country, the Ministry of Health could establish a technical ZDU similar to that in Kenya (or an OHCD similar to that in Tanzania), with personnel deputed from each of the ministerial units to ensure mutual exchange of information and coordination during a major crisis that could span multiple ministries. A common data platform could also be developed by the office of the Principal Scientific Advisor (PSA), which is used by all the ministries and departments across the country, with the request not to develop stand-alone data systems. Admittedly, the creation of these multiple units, on the face of it, lacks the elegance associated with a single all-encompassing unit directly engaging with all One Health-related matters. In theory, such a unit should be able to act as the perfect clearinghouse of all information and the manager of all risks. In practice, even in the, arguably simpler, domain of financial services, this has not proven to be a durable model, as the now famous breakup of the Financial Services Authority in the UK demonstrated [63]. In the Indian context as well, these ideas have also been debated and, ultimately, not pursued [64].

The third relates to benefiting from their unique position within the Indian context and adding a One Health officer within the office of each District Collector and ensuring that all the officers of the Indian Administrative Service receive training in One Health at their training institute in Mussoorie.

## 7 Recommendations

Given the inherent complexity associated with One Health and the number of links it has to almost every sphere of human activity, as the experience of many countries that have attempted to grapple with this challenge demonstrates, it has been difficult to develop an organizational structure within the government that is effectively able to address the issues involved. If it is positioned too high (as in the case of Rwanda), it is very quickly disconnected from ground realities and risks becoming irrelevant. If placed within a particular ministry, it functions effectively as long as the issues involved stay within the direct span of control of the ministry, but as soon as intersectoral collaboration is called for (as in the case of India), this approach becomes less effective. A lean technical unit established by multiple ministries (such as in the case of the ZDU in Kenya and the OHCD in Tanzania) has proven to be effective, but only in its response to crises and not for longer-term issues. However, if all these insights are taken together, it may be possible to develop an effective strategy that combines the best elements of each approach. In the Indian context, such a strategy would involve the establishment of multiple units mentioned in Table 7, each with a carefully defined role. The units mentioned in Table 7 are at the national level in India and could also be replicated at the state level. However, given how different each state is, these structures may need to be suitably modified.

**Table 7:** One Health Governance Structures for India.

Unit Name	Potential Location	Functions
One Health Science Unit	Office of the Principal Scientific Advisor	Ensuring that the country has the scientific, laboratory, and surveillance capacity to identify emerging pathogens; coordinate with the Education and Health Ministries to ensure that One Health is prominently featured in undergraduate and graduate curricula.
One Health Strategy Unit	NITI Aayog	Working closely with the other arms of NITI Aayog, various ministries, and state governments to ensure that the overall development strategy followed by them is consistent with One Health.
Ricketssial & Protozoal Unit	Ministry of Housing and Urban Affairs	Given the close links of these diseases with, among other things, rodents, poor sanitation, and poorly drafted and implemented building codes, the focus of this unit would be to ensure that the possibilities of cross-over and transmission are minimised in urban areas.
Bacterial Unit	Ministry of Agriculture	Ensuring that the possibility of crossover and transmission of bacterial pathogens is minimised during agricultural activities.
Viral Unit (Human Health)	Ministry of Travel & Tourism	Given the strong transmission links with travel, domestic and international, this unit would focus on the establishment of routine surveillance at transit points and rapid emergency response during a crisis.
One Health Unit (Wild Animal Health)	Ministry of Environment & Forests	The focus of this unit would be active surveillance of emerging pathogens, developing longer-term strategies to minimise human-wild-animal contact, and ensuring the health of wild animals.
One Health Unit (Farm Animal Health)	Ministry of Animal Husbandry, Dairying and Fisheries	The focus of this unit would be active surveillance of emerging pathogens and the development of long-term strategies for safe practises.
One Health Unit (Tribal Health)	Ministry of Tribal Affairs	Given the close proximity to wildlife and forest of tribal communities, the focus of this unit would be active surveillance of emerging pathogens, on the health of tribal communities, and the possibility of zoonotic spillovers.
Zoonotic Disease Unit	Ministry of Health & Family Welfare	This unit could act as a lean information exchange unit between all the focused ministerial units; it could also ensure that all essential public health functions are carried out with high fidelity throughout the country.
District One Health Officer	Office of the District Magistrate	This individual would act as a clearing house for One Health information and would keep the District Magistrate briefed. During a crisis, this individual would act to convene all the relevant departments at the district level on behalf of the District Magistrate and keep track of all associated activities. The day-to-day work of this individual would need to be carefully structured so that even in times when there are no One Health crises, continuous progress is being made within the district on multiple aspects of One Health.

## Declaration of Competing Interest

The authors declare that they have no known competing financial interests or personal relationships that could have appeared to influence the work reported in this paper.

## Data Availability

No data was used for the research described in the article.
